# Analyzing the effectiveness and safety of arthroscopic suture anchor repair for anterior talofibular ligament in patients with ankle instability

**DOI:** 10.1371/journal.pone.0324602

**Published:** 2025-07-07

**Authors:** Zhaole Xu, Qian Li, Xuan Ding, Yiqiong Nie, Ning Chen

**Affiliations:** Department of Orthopaedics, The First Traditional Chinese Medicine Hospital of Changde City, Changde City, Hunan Province, China; Policlinico Universitario A. Gemelli IRCCS - Universita Cattolica del Sacro Cuore Roma, ITALY

## Abstract

**Background:**

Ankle instability, often secondary to anterior talofibular ligament (ATFL) injury, poses significant challenges in orthopedic management. This study aimed to evaluate the effectiveness and safety of arthroscopic suture anchor repair for ATFL in patients with ankle instability.

**Methods:**

This retrospective study spanned from January 2022 to February 2024 and involved 58 patients who underwent arthroscopic repair and 60 patients who underwent the open Broström procedure. Baseline characteristics, surgical outcomes, and functional assessments utilizing the Ankle Hindfoot Scale (AOFAS) were compared between the two groups. Additionally, talar tilt angle and anterior translation distance were measured using anteroposterior and lateral ankle radiographs.

**Results:**

Both groups showed significant improvements in AOFAS scores post-treatment (p < 0.001). The observation group had shorter surgical durations, reduced blood loss, and quicker recovery times compared to the control group (p < 0.001). Talus tilt angle and anterior translation distance decreased significantly in both groups postoperatively (p < 0.001). Arthroscopic repair demonstrated superior outcomes in terms of AOFAS scores and surgical parameters.

**Conclusions:**

Arthroscopic suture anchor repair is an effective and safe option for managing ankle instability, offering procedural efficiency, reduced blood loss, and favorable functional outcomes. Further research is needed to validate these findings and compare arthroscopic techniques with conventional open procedures.

## 1. Introduction

Ankle instability, a common orthopedic condition, presents significant challenges in both diagnosis and management. Among the various structures contributing to ankle stability, the anterior talofibular ligament (ATFL) plays a crucial role, and its injury often leads to recurrent instability episodes [1,2]. Traditional open surgical techniques for ATFL repair have shown efficacy but may be associated with drawbacks such as extensive soft tissue disruption and prolonged recovery times [3]. Accurate diagnosis of chronic ankle instability is fundamental to optimizing patient selection and evaluating surgical outcomes. Clinically, the assessment of ligamentous laxity and functional instability primarily relies on physical examination tests, such as the anterior drawer test and the talar tilt test, which have been rigorously evaluated for their diagnostic accuracy. Recent studies have validated these tests, demonstrating their reliability in quantifying mechanical instability. In addition, imaging modalities, including stress radiography and magnetic resonance imaging (MRI), provide complementary structural information that further refines the diagnostic process. This multimodal diagnostic approach not only enhances the accuracy of detecting ATFL injuries but also facilitates a more objective evaluation of the severity of instability [4–6].

In recent years, arthroscopic suture anchor repair has emerged as a promising alternative for ATFL reconstruction in patients with ankle instability. Several high-level clinical studies have directly compared arthroscopic suture anchor repair with traditional open techniques for ATFL reconstruction. These studies have comprehensively assessed multiple outcomes, including functional recovery, complication rates, and patient-reported satisfaction. Certain randomized controlled trials have reported that arthroscopic repair may result in reduced intraoperative soft tissue disruption, diminished postoperative pain, and faster rehabilitation, while demonstrating efficacy comparable to open repair regarding joint stability and the recurrence of instability [7,8]. This minimally invasive approach offers several potential advantages over traditional open surgery, including reduced surgical trauma, improved visualization of the joint, and enhanced precision in ligament repair [9,10]. By utilizing small incisions and specialized instrumentation, arthroscopic techniques enable targeted repair of the ATFL while minimizing damage to surrounding tissues. The effectiveness and safety of arthroscopic suture anchor repair for ATFL injuries have garnered increasing attention within the orthopedic community [11,12]. Numerous studies have investigated its outcomes in terms of functional recovery, recurrence rates, and patient satisfaction. These studies have reported favorable results, with many demonstrating comparable or superior outcomes to open surgical techniques. Moreover, arthroscopic approaches have been associated with shorter postoperative rehabilitation periods and earlier return to activities of daily living, thus potentially reducing the overall burden on patients and healthcare systems [13,14].

Despite the growing body of evidence supporting the use of arthroscopic suture anchor repair, several important considerations warrant further exploration. Variability in patient demographics, injury severity, and surgical techniques across studies may influence outcomes and complicate result interpretation. Additionally, long-term follow-up data are essential to assess the durability of repair and the incidence of late complications such as osteoarthritis and joint stiffness. Given the potential benefits of arthroscopic techniques and the need for comprehensive understanding of their effectiveness and safety profile, this study aims to analyze the outcomes of arthroscopic suture anchor repair for ATFL injuries in patients with ankle instability.

## 2. Methods

### 2.1 Study design

A retrospective study was conducted at our institution to assess the effectiveness and safety of arthroscopic suture anchor repair for anterior talofibular ligament (ATFL) in patients with ankle instability. The study period spanned from January 2022 to February 2024. A total of 58 patients who underwent arthroscopic suture anchor repair were included in the observation group for analysis. For comparative analysis, a control group of 60 patients who underwent the open Broström procedure with anchor fixation during the same period was established to ensure comparability between the cohorts. Informed consent was obtained from all subjects and/or their legal guardian(s). The study’s protocols and methods were rigorously reviewed by our hospital’s ethics committee and conducted in accordance with established guidelines and regulations. Adhering strictly to the Declaration of Helsinki, the study ensured the ethical treatment of participants. All data was confidentially managed and anonymized to maintain privacy.

### 2.2 Inclusion and exclusion criteria

#### 2.2.1 Inclusion criteria.

1) Patients with clinically diagnosed chronic ankle instability attributed to an isolated ATFL injury, confirmed by arthroscopic examination demonstrating damage at the fibular attachment of the ATFL.
2) Patients undergoing either arthroscopic suture anchor repair or the open Broström procedure for ATFL reconstruction, performed by experienced orthopedic surgeons.
3) Availability of comprehensive preoperative and postoperative clinical data, including standardized functional scores, imaging studies, and detailed physical examination findings.
4) Patients aged between 18 and 60 years.
5) Written informed consent obtained from all participants.

#### 2.2.2 Exclusion criteria.

1) Patients with concomitant injuries to other lateral ankle ligaments (e.g., calcaneofibular or posterior talofibular ligaments) requiring additional surgical interventions.
2) History of previous ankle surgeries or significant ankle trauma that could potentially affect current treatment outcomes.
3) Patients with systemic or metabolic disorders, such as diabetes mellitus or rheumatoid arthritis, known to impact wound healing or bone metabolism.
4) Patients with neurological disorders or conditions affecting lower limb motor function.
5) Patients who were unable to adhere to the specified follow-up protocol, which required a minimum duration of 3 months, were excluded from the study.

### 2.3 Observation group arthroscopic suture anchor repair intervention protocol

In the observation group, patients underwent arthroscopic suture anchor repair under general anesthesia. The patients were positioned supine with a pneumatic tourniquet applied to the thigh of the affected side to maintain ankle dorsiflexion neutral position. An incision was made along the anterior margin of the tibialis anterior tendon for visualization (medial midline approach), with an additional incision made approximately 1.5 cm anterior to the tip of the fibula for operative access (supplementary anterior lateral approach). Arthroscopic examination was performed to assess the extent of damage to intra-articular structures including cartilage and ATFL. Surgical interventions included debridement of osteophytes, synovial tissue clearance, and repair of articular cartilage defects, followed by site-specific suture repair upon identification of the injured area.

The procedure consisted of two main steps: (1) anchor insertion, where a 2.9 mm diameter absorbable bone anchor with two strands of size 2 ULTRABRAID™ ultra-strong non-absorbable suture was inserted at the fibular attachment site of the ATFL; (2) passage of the two suture strands through the ATFL, followed by knot tying and fixation to the fibula. The technique involved inserting a needle, carrying a nylon loop, approximately 1 cm below the supplementary anterior lateral approach, directing it through the skin, subcutaneous tissue, into the ankle joint space, passing through the ATFL from bottom to top, gripping the nylon loop with forceps and withdrawing it, guiding one suture strand through the ATFL via the nylon loop and extracting it from the supplementary anterior lateral approach, repeating the process for the second suture strand to reinforce the extensor retinaculum, securing the sutures with knots at the fibular attachment site of the ATFL, and closing the incision.

### 2.4 Control group open broström anchor fixation intervention protocol

Patients in the control group underwent open Broström anchor fixation surgery under general anesthesia. The patients were positioned supine with a pneumatic tourniquet applied to the affected thigh to ensure hemostasis. An arthroscopic examination was first performed to assess intra‐articular structures, including cartilage and the ATFL. Subsequent surgical interventions encompassed debridement of osteophytes, clearance of synovial tissue, and repair of any detected articular cartilage defects, followed by targeted suture repair of the injured area. A modified open Broström approach was then implemented through a 3–4 cm incision made anterior to the distal fibula, with careful, layer-by-layer dissection to separate the ATFL and extensor retinaculum. A double-stranded absorbable bone anchor (Smith+Nephew) was inserted at the fibular attachment site of the ATFL. The suture strands were subsequently secured to the fibula, and reinforcement of the extensor retinaculum was performed prior to closure of the incision.

Both the arthroscopic and open repairs were performed by the same surgical team. Both groups underwent a standardized postoperative rehabilitation protocol to ensure consistency in functional recovery. This protocol involved an initial immobilization phase in a below-knee cast for 2 weeks, during which weight-bearing was restricted. After cast removal, a structured physical therapy program was initiated, comprising early range-of-motion exercises, progressive strengthening exercises, and proprioceptive training. This regimen was maintained for the subsequent weeks, with treatment outcomes assessed at 3 months postoperatively.

### 2.5 Data collection

These parameters included surgical duration, intraoperative blood loss, and the duration until patients resumed normal activities postoperatively. Baseline physical activity was assessed using the Tegner Activity Scale. Additionally, functional outcomes were evaluated using the Ankle Hindfoot Scale (AOFAS) at baseline and at the 3-month follow-up. The AOFAS score, with a total range of 0 to 100, comprehensively assesses joint alignment, ankle dorsiflexion, stability, walking ability, and pain, with higher scores indicating better functional outcomes. Furthermore, objective measures of ankle stability were obtained through radiographic analysis. Standardized anteroposterior and lateral stress radiographs were acquired under controlled conditions both preoperatively and at the 3-month postoperative follow-up. Using calibrated digital imaging software, measurements of the talar tilt angle and talar anterior translation distance were performed to assess the degree of anatomical restoration. Lastly, occurrences of postoperative complications were documented and analyzed to evaluate the safety profile of the respective treatment modalities.

### 2.6 Statistical analysis

Statistical analyses were conducted meticulously utilizing SPSS software (Version 27.0). Initially, the data were categorized into quantitative or categorical variables, with assessments of normality performed to determine their distribution patterns. Quantitative variables conforming to a normal distribution underwent inter-group comparison using independent sample t-tests, with results reported as mean ± standard deviation. Conversely, non-normally distributed quantitative variables were summarized using median and interquartile ranges (M[P25, P75]), and between-group comparisons were conducted using the Mann-Whitney U test. Categorical variables were presented as frequencies and percentages, and associations among these variables were assessed using Chi-square (χ^2^) tests. All statistical tests were two-tailed, and a significance level of p < 0.05 was utilized to determine statistical significance.

## 3. Results

### 3.1 Comparison of baseline characteristics

The control group comprised 35 male and 25 female patients, with ages ranging from 21 to 56 years and a mean age of 28.96 ± 3.6 years. The interval between injury and surgery ranged from 1 to 6 months, with a mean duration of 3.96 ± 0.59 months. In the observation group, there were 31 male and 27 female patients, with ages ranging from 19 to 51 years and a mean age of 26.98 ± 3.56 years. The interval between injury and surgery ranged from 1 to 6 months, with a mean duration of 3.78 ± 0.66 months. There were no statistically significant differences in baseline characteristics between the two groups (p > 0.05), indicating comparability. In the control group, baseline physical activity was assessed using the Tegner Activity Scale, yielding a mean score of 5.1 ± 1.0, while the observation group recorded a comparable mean score of 5.2 ± 1.1 (p > 0.05). Moreover, the degree of ligament laxity was evaluated by quantifying the anterior drawer test displacement. The control group exhibited a mean displacement of 5.8 ± 1.2 mm, whereas the observation group had a mean displacement of 5.9 ± 1.3 mm (p > 0.05). The demographic characteristics of patients, including gender distribution, age distribution, and duration from injury to surgery, were well-balanced between the control and observation groups, ensuring homogeneity and minimizing potential confounding factors in subsequent analyses.

### 3.2 Comparison of AOFAS Scores before and after treatment between

The AOFAS scores significantly improved in both the control and observation groups after treatment. Before treatment, the mean AOFAS score was similar between the two groups, with no significant difference observed (Control: 64.18 ± 7.00, Observation: 62.33 ± 6.07). However, after 3 months of treatment, the mean AOFAS score increased substantially in both groups, indicating enhanced ankle and hindfoot function (Control: 78.49 ± 6.48, Observation: 84.72 ± 7.33). Statistical analysis revealed significant differences in the mean AOFAS scores before and after treatment within each group (Control: t = 11.025, p < 0.001; Observation: t = 18.202, p < 0.001). Additionally, the improvement in AOFAS scores was more pronounced in the observation group compared to the control group, as evidenced by the higher post-treatment scores and larger t-value. These findings suggest that both arthroscopic suture anchor repair and open Broström anchor fixation procedures effectively improve ankle function, with the observation group demonstrating superior outcomes (Table 1).

**Table 1:** Comparison of AOFAS Scores Before and After Treatment between Control and Observation Groups (scores, mean ± SD).

Group	Before Treatment	After 3 Months	t Value	p Value
Control	64.18 ± 7.00	78.49 ± 6.48	11.025	< 0.001
Observation	62.33 ± 6.07	84.72 ± 7.33	18.202	< 0.001
t Value	1.915	7.759	–	–
p Value	0.059	< 0.001	–	–

AOFAS – Ankle Hindfoot Scale.

### 3.3 Surgical outcomes comparison between control and observation groups

The comparison of surgical outcomes between the control and observation groups revealed significant differences. Specifically, the mean surgical duration was 36.63 ± 4.80 minutes in the control group and 22.60 ± 5.41 minutes in the observation group (t = 14.88, p < 0.001). Intraoperative blood loss averaged 28.96 ± 5.19 mL for the control group versus 10.78 ± 2.05 mL for the observation group (t = 21.25, p < 0.001). Additionally, the time to recovery was 8.04 ± 2.01 days in the control group compared to 6.02 ± 1.68 days in the observation group (t = 5.656, p < 0.001) (Table 2). These findings suggest that patients in the observation group experienced favorable surgical outcomes, characterized by shorter surgical duration, reduced intraoperative blood loss, and quicker recovery times, compared to those in the control group.

**Table 2:** Comparison of Surgical Duration, Intraoperative Blood Loss, and Time to Recovery between Control and Observation Groups.

Group	Surgical Duration (min)	Intraoperative Blood Loss (mL)	Time to Recovery (days)
Control	36.63 ± 4.80	28.96 ± 5.19	8.04 ± 2.01
Observation	22.60 ± 5.41	10.78 ± 4.05	6.02 ± 1.68
t Value	14.88	21.25	5.656
p Value	< 0.001	< 0.001	< 0.001

### 3.4 Comparison of talus tilt angle changes before and after treatment in control and observation groups.

The control group underwent open Broström procedure, while the observation group underwent arthroscopic suture anchor repair. Our findings revealed a substantial reduction in talus tilt angle in both groups after treatment. Specifically, the control group experienced a mean decrease from 9.18 ± 1.88 degrees before treatment to 3.29 ± 1.56 degrees after 3 months (t = 11.025, p < 0.001). Similarly, the observation group exhibited a significant decrease from 9.21 ± 1.96 degrees before treatment to 3.10 ± 1.69 degrees after 3 months (t = 18.202, p < 0.001). Statistical analysis confirmed the significance of these changes, with p-values well below 0.001. These results suggest that both open Broström procedure and arthroscopic suture anchor repair effectively reduce talus tilt angle and enhance ankle stability in patients with ankle instability (Table 3).

**Table 3:** Comparison of Talus Tilt Angle before and after Treatment between Control and Observation Groups (mean ± SD).

Group	Before Treatment	After 3 Months	t Value	p Value
Control	9.18 ± 1.88	3.29 ± 1.56	11.025	< 0.001
Observation	9.21 ± 1.96	3.10 ± 1.69	18.202	< 0.001
t Value	0.079	0.034	–	–
p Value	0.937	0.973	–	–

### 3.5 Comparison of anterior translation distance before and after treatment in control and observation groups.

Subsequent to treatment, significant reductions in anterior translation distance were observed in both groups. Specifically, the control group experienced a mean decrease from 8.36 ± 1.98 to 3.08 ± 1.89 (t = 13.025, p < 0.001), while the observation group demonstrated a reduction from 8.58 ± 2.01 to 3.01 ± 1.83 (t = 12.986, p < 0.001). Statistical analysis confirmed the significance of these reductions, with p-values below 0.001(Table 4). These outcomes underscore the effectiveness of the treatment modalities, including the open Broström procedure in the control group and arthroscopic suture anchor repair in the observation group, in ameliorating anterior translation distance among individuals with ankle instability.

**Table 4:** Comparison of Anterior Translation Distance Before and After Treatment between Control and Observation Groups (mean ± SD).

Group	Before Treatment	After 3 Months	t Value	p Value
Control	8.36 ± 1.98	3.08 ± 1.89	13.025	< 0.001
Observation	8.58 ± 2.01	3.01 ± 1.83	12.986	< 0.001
t Value	0.192	0.367	–	–
p Value	0.851	0.703	–	–

### 3.6 Post-hoc power analysis.

The post-hoc power analysis, conducted using a weighted approach, revealed that the statistical power of the study was exceptionally high due to the large effect sizes observed for most of the primary endpoints, particularly surgical duration, intraoperative blood loss, recovery time, and radiographic measures. The weighted effect size total was substantially large, resulting in a power well above 80%, indicating that the sample size was sufficient to detect significant differences between the treatment groups.

## 4. Discussion

Ankle instability, characterized by recurrent lateral ankle sprains and a sensation of giving way, is a common musculoskeletal condition that significantly impacts function and quality of life [15]. The anterior talofibular ligament (ATFL) plays a crucial role in restraining excessive inversion and anterior translation of the talus relative to the tibia, with injury or laxity of the ATFL being a primary contributor to recurrent sprains and instability [16,17]. Traditional surgical techniques, such as the open Broström procedure, are effective in restoring ankle stability but are associated with prolonged surgical duration, extensive soft tissue dissection, and a higher risk of wound complications. In contrast, arthroscopic suture anchor repair has emerged as a minimally invasive alternative, offering several potential advantages, including targeted ligament repair, reduced morbidity, and preserved soft tissue integrity [18,19]. However, despite the growing adoption of arthroscopic repair, limited evidence exists comparing its effectiveness and safety with open procedures [20,21]. Our study is novel in that it integrates both subjective (functional recovery scores) and objective (radiographic parameters such as talar tilt angle and anterior translation distance) outcome measures to provide a comprehensive evaluation of postoperative joint stability. By directly comparing the outcomes of arthroscopic suture anchor repair with the open Broström procedure, this study offers new insights into the relative advantages of the arthroscopic approach in terms of surgical efficiency, recovery time, and functional outcomes in patients with chronic ankle instability.

By evaluating clinical outcomes, including ankle stability, functional improvement, and complication rates, we sought to provide valuable insights into the utility of arthroscopic techniques in the management of ankle instability [22,23]. Our study evaluated the effectiveness of arthroscopic suture anchor repair in managing ankle instability, particularly in comparison to the traditional open Broström procedure. The findings underscored several notable advantages associated with arthroscopic techniques, highlighting its potential as a preferred surgical approach for ankle stabilization. Arthroscopic suture anchor repair offers several distinct advantages over open surgical techniques. One of the key benefits observed in our study was the significantly shorter surgical duration associated with arthroscopic procedures compared to open surgery. The minimally invasive nature of arthroscopic techniques allows for precise visualization and targeted repair of ligamentous structures, resulting in reduced operative time. This efficiency is crucial for optimizing surgical workflow and minimizing patient morbidity [24].

Another notable advantage of arthroscopic suture anchor repair is the significantly reduced intraoperative blood loss observed in our study. The minimally invasive nature of arthroscopic procedures minimizes tissue trauma and vascular injury, leading to decreased intraoperative bleeding. This reduction in blood loss not only contributes to improved surgical safety but also facilitates clearer visualization of the operative field, enhancing surgical precision and efficacy [25,26]. Our findings also demonstrated significantly shorter recovery times associated with arthroscopic suture anchor repair compared to traditional open techniques. The less invasive nature of arthroscopic procedures results in reduced postoperative pain, swelling, and tissue damage, facilitating faster recovery and rehabilitation. Quicker recovery times are advantageous for patients, allowing for earlier return to activities of daily living and reduced healthcare resource utilization.

In addition to procedural advantages, arthroscopic suture anchor repair yielded favorable clinical outcomes in terms of ankle stability and functional improvement. The significant reductions in talus tilt angle and anterior translation distance observed post-treatment underscored the effectiveness of arthroscopic techniques in restoring normal joint biomechanics and enhancing ankle stability. These improvements are crucial for preventing recurrent ankle sprains and maintaining long-term joint integrity. The findings of our study have important clinical implications for the management of ankle instability [27,28]. Arthroscopic suture anchor repair represents a valuable alternative to traditional open techniques, offering several advantages, including shorter surgical duration, reduced intraoperative blood loss, and quicker recovery times. Surgeons may consider incorporating arthroscopic approaches into their treatment algorithms for ankle instability, particularly in cases where procedural efficiency and patient outcomes are paramount. However, it is important to note that although the improvement in AOFAS scores was statistically significant between the two groups—with the observation group exhibiting a higher post-treatment mean score—a difference of 5–10 points may not necessarily translate into a clinically significant improvement. Prior studies indicate that the minimal clinically important difference (MCID) for the AOFAS score can vary, and in some contexts, changes within this range may not be readily perceptible to patients in terms of functional outcomes. Similar considerations apply to the other statistically significant differences observed in our study. Therefore, while our findings confirm that both surgical approaches effectively enhance ankle function, the clinical relevance of the observed intergroup difference should be interpreted with caution.

Several limitations inherent to our study design warrant consideration. First, the retrospective design may have introduced selection bias and uncontrolled confounding factors that might have influenced the observed outcomes. Second, future studies with larger sample sizes and standardized rehabilitation protocols will enhance the ability to draw more definitive conclusions regarding the comparative effectiveness of arthroscopic versus open repair techniques. Third, the analysis is based solely on short-term outcomes, thereby necessitating longer-term follow-up to fully assess the durability of the treatment effects. Lastly, the absence of a control group treated with alternative interventions precludes direct comparisons of the efficacy of arthroscopic suture anchor repair with other techniques. Future research should also explore procedures targeting the CFL or peroneal tendons, as well as combined ATFL and CFL repair, to provide a more comprehensive understanding of lateral ankle instability. Prospective, multicenter studies with larger cohorts and extended follow-up will further elucidate the effectiveness and safety of arthroscopic approaches in chronic ankle instability management.

## 5. Conclusions

In conclusion, our study highlights the efficacy and advantages of arthroscopic suture anchor repair in the management of ankle instability. The procedural efficiency, reduced intraoperative blood loss, quicker recovery times, and favorable clinical outcomes associated with arthroscopic techniques underscore its potential as a preferred surgical approach for ankle stabilization. Further research and long-term follow-up studies are warranted to validate these findings and elucidate the comparative effectiveness of arthroscopic versus open surgical interventions in the management of ankle instability.
